# Do CAD/CAM restorative materials respond differently to coffee thermocycling and simulated toothbrushing?

**DOI:** 10.1007/s00784-026-06836-5

**Published:** 2026-03-23

**Authors:** Irem Sozen Yanik, Dilara Sahin Hazir, Guliz Aktas, M. Baris Guncu

**Affiliations:** Department of Prosthodontics, Faculty of Dentistry, Hacettepe University, Sihhiye, Ankara, 06100 Turkey

**Keywords:** CAD/CAM restorative materials, Color stability, Surface roughness, Coffee staining, Toothbrushing abrasion

## Abstract

**Objectives:**

This laboratory study aimed to investigate the effects of toothbrushing with different toothpaste formulations on the color stability and surface roughness of CAD/CAM restorative materials following coffee-staining thermocycling.

**Materials and methods:**

A total of 160 specimens were prepared from four restorative materials: IPS e.max CAD, CEREC Tessera, Shofu Block HC, and VITA Enamic (*n* = 40 each). Following initial measurements, specimens underwent thermocycling in a coffee solution (5000 cycles) to simulate staining. Each group was subsequently divided and brushed with either a whitening toothpaste containing hydrated silica and hydrogen peroxide or a non-whitening toothpaste containing hydrated silica only. A total of 10,000 brushing cycles were performed under standardized conditions. Color stability was assessed using ΔE₀₀ values, and surface roughness was quantified using the Ra parameter at baseline, after thermocycling, and after brushing. Data were analyzed using Kruskal–Wallis and Mann–Whitney U tests with the significance threshold set at *p* < 0.05. SEM analysis was conducted on one randomly selected specimen per group (x1000).

**Results:**

Statistically significant differences in color stability, as assessed by ΔE₀₀ values, were observed among the tested materials (*p* < 0.001). Resin-based groups, particularly Shofu Block HC, exhibited the greatest reduction in color stability after coffee thermocycling and subsequent brushing (ΔE₀₀ = 1.069, *p* < 0.001), whereas glass-ceramics (IPS e.max CAD and CEREC Tessera) demonstrated superior color stability (ΔE₀₀ ≤ 0.520). Surface roughness increased significantly in resin-containing materials (VITA Enamic ΔRa = 0.036; Shofu Block HC ΔRa = 0.020) compared with glass-ceramics (*p* < 0.001). No significant differences were detected between whitening and non-whitening toothpastes for either parameter across all groups (*p* > 0.05). Ra changes were also concluded by SEM.

**Conclusion:**

The performance of restorative materials under staining and mechanical aging stressors is material-dependent. Glass-ceramics displayed superior color stability after coffee exposure and greater resistance to brushing-related surface degradation, whereas resin-containing materials were more vulnerable. The choice of toothpaste formulation did not significantly affect color stability or surface characteristics, suggesting that intrinsic material properties play a more critical role in long-term esthetic outcomes.

**Clinical relevance:**

Selection of restorative materials plays a decisive role in ensuring the long-term esthetic success of dental restorations. Since intrinsic material properties strongly influence color stability and resistance to surface degradation, clinicians should carefully consider material choice during treatment planning to achieve durable and predictable outcomes.

## Introduction

To meet esthetic and functional demands, a wide range of ceramic and resin-based composite materials have been developed [1, 2]. Owing to their excellent biocompatibility, mechanical strength, resin nanoceramics and polymer-infiltrated ceramic network (PICN) materials have been widely utilized in restorative dentistry over the past decade [3, 4]. The long-term clinical success of indirect dental restorations critically depends on accurate shade matching and the preservation of surface integrity [5, 6].

Surface properties are influenced by both intrinsic material characteristics and extrinsic factors such as water absorption and the adsorption of pigments from common dietary substances [3, 7, 8]. Previous studies have shown that CAD/CAM resin-based and glass-ceramic materials exhibit material-dependent differences in color stability when exposed to staining agents, with coffee being identified as one of the most chromogenic solutions, particularly affecting the color stability of resin nanoceramics compared to glass ceramics [9–11]. Factors related to colored beverage consumption, along with material aging, play a significant role in the color stability of restorations [3, 12]. Coffee consumption has markedly increased worldwide, and it is well recognized as one of the dietary agents with a strong potential to adversely affect the esthetic appearance of teeth and restorative materials. Coffee has been shown to markedly impact the color stability of resin-based materials through the interaction of its yellow, low-polarity molecules with the polymer matrix [7, 13]. Particularly, the intake of hot coffee has been reported to intensify staining effects, thereby exacerbating color instability in restorative surfaces [14, 15].

Recent in vitro findings have confirmed that lithium disilicate and zirconia-reinforced lithium silicate ceramics display higher color stability than polymer-infiltrated and resin-based materials after thermocycling and coffee immersion, due to their lower water sorption and more stable glass networks [10, 16, 17].

Staining is not the only factor that affects the performance of restorative materials; toothbrushing also plays a critical role, as it influences both color stability and surface roughness. This is mainly attributed to the mechanical abrasion caused by toothbrush bristles and the chemical abrasivity of dentifrices. Whitening toothpastes, which are popular for their cosmetic benefits, frequently contain abrasive agents (e.g., hydrated silica, calcium carbonate, perlite) and bleaching compounds (e.g., hydrogen peroxide) [18–20]. While these formulations enhance cleaning efficacy and stain removal, they may also contribute to surface degradation and wear over time [5].

According to recent evidence, abrasive and whitening toothpastes significantly increase surface roughness and microwear on resin nanoceramics and polymer-infiltrated ceramics compared with glass-ceramic materials [21–24]. The magnitude of surface damage is closely related to the abrasivity index, particle morphology, and the brushing force used during simulated toothbrushing [21]. Moreover, hybrid CAD/CAM materials demonstrate higher susceptibility to surface roughening and gloss loss than lithium disilicate ceramics following prolonged brushing cycles [22, 24].

Recent studies have increasingly focused on how restorative materials respond to aging-related stressors under simulated clinical conditions [3, 5]. Both toothbrushing and thermocycling have been shown to significantly affect the color stability and surface quality of restorative materials, with whitening toothpastes leading to more pronounced surface degradation compared to conventional formulations [5, 25, 26]. Polymer-infiltrated ceramic networks (PICNs) and resin nanoceramics are particularly susceptible to reduced color stability [3], and brushing simulations have revealed distinct, material-dependent variations in surface gloss and roughness, especially after extended mechanical aging cycles [27]. Building on these findings, previous studies have demonstrated that simulated toothbrushing can cause measurable optical and surface changes in restorative materials. While long-term brushing produces minor but clinically acceptable alterations in ceramics, hybrid and resin-based composites exhibit more evident surface deterioration. Whitening formulations containing abrasive or oxidizing agents further intensify surface roughness and color instability, particularly in resin nanoceramics compared with zirconia-reinforced lithium silicate ceramics [21, 23].

This laboratory study aimed to investigate the effects of toothbrushing with different toothpaste formulations on the color stability and surface roughness of CAD/CAM restorative materials following coffee-staining thermocycling. Based on the current knowledge, the null hypothesis was that no significant differences would be observed in color stability and surface roughness among the tested materials following coffee immersion and brushing with different toothpaste formulations.

## Materials and methods

Sample size calculation was performed using the G*Power software package (version 3.1.9.7, Heinrich-Heine-Universitat Dusseldorf, Germany). Based on a one-way ANOVA test, with a medium effect size (f = 0.28), a significance level of 0.05 (α = 5%), and a statistical power of 80%, the required minimum sample size was determined to be 36 specimens per group, for a total of 144 specimens [28]. Considering the possibility of specimen loss or experimental error, the total sample size was increased to 160 specimens to ensure adequate statistical reliability.

### Preparation of specimens

A total of 160 square-shaped specimens (6 mm × 6 mm × 2 mm) were prepared from four different restorative materials (*n* = 40 each): IPS E.max CAD (IE; Ivoclar Vivadent AG, Schaan, Liechtenstein), CEREC Tessera (TS; Dentsply Sirona, York, PA, USA), Shofu Block HC (SH; Shofu Dental Corp., Kyoto, Japan), and VITA Enamic (VE; Vita Zahnfabrik, Bad Säckingen, Germany). The specimens were sectioned using a low-speed diamond blade mounted on a precision cutting machine (Isomet 15LC; Buehler, Lake Bluff, IL, USA). The composition of the materials is summarized in Table 1.

**Table 1:** Evaluated materials and respective acronym, type, composition, and shade

Material, Acronym, Type	Filler	Polymer	Shade
IPS E.max CAD (IE), Lithium disilicate glass ceramic	57–80% SiO₂, 11–19% Li₂O, other oxides	–	A2 LT
Tessera (TS), Zirconia-reinforced lithium silicate glass ceramic	56–64% SiO₂, 8–12% ZrO₂, 15–21% Li₂O, 1–8% other oxides	–	A2 MT
Shofu Block HC (SH), Hybrid ceramic / Resin nanoceramic	61% ZrSiO₄-based glass and SiO₂	UDMA, TEGDMA	A2 LT
VITA Enamic (VE), Polymer-infiltrated ceramic network (PICN)	58–63% SiO₂, 20–23% Al₂O₃, 9–11% Na₂O, 4–6% K₂O, 0.5–2% B₂O₃, < 1% ZrO₂, < 1% CaO	14% UDMA, TEGDMA	1M2 T

Lithium disilicate and lithium silicate glass ceramic specimens were crystallized in a ceramic furnace (Programat EP5000, Ivoclar Vivadent AG, Schaan, Liechtenstein) according to the manufacturers’ recommended firing schedules. All specimens were sequentially pre-polished under continuous water-cooling using silicon carbide abrasive papers of #180, #320, #400, and #600 grit (Struers GmbH, Ballerup, Denmark) on a grinding machine (EcoMet 6, Buehler, USA) to obtain standardized flat surfaces prior to mechanical polishing. Following pre-polishing, material-specific chairside mechanical polishing protocols were applied in accordance with the manufacturers’ instructions. Glass-ceramic specimens were mechanically polished using diamond-impregnated rubber polishing instruments (DIASYNT PLUS RA, EVE Ernst Vetter GmbH, Pforzheim, Germany) in a low-speed handpiece without water cooling, as recommended by the manufacturer. Polishing was performed sequentially at a rotational speed of up to 12,000 rpm, using light manual pressure, until a smooth and uniform surface was obtained. Polymer-infiltrated ceramic network (PICN) and resin nanoceramic specimens were polished using a diamond-impregnated polishing system specifically indicated for composite and hybrid ceramics (DIACOMP PLUS RA set, EVE Ernst Vetter GmbH, Pforzheim, Germany). Polishing was carried out in a low-speed handpiece under water cooling, at a rotational speed of up to 8000 rpm, in accordance with the manufacturer’s instructions, using sequential polishing steps and light manual pressure. After mechanical polishing, a final high-shine polishing step was applied. For glass-ceramic materials, a diamond polishing paste (DIAPOL^®^ Paste, EVE Ernst Vetter GmbH, Pforzheim, Germany) was applied mechanically using a nylon polishing brush at low speed, in accordance with the manufacturer’s recommendations. For PICN and resin nanoceramic specimens, a polishing paste (DIACOMP polishing paste, EVE Ernst Vetter GmbH, Pforzheim, Germany) was used in the same manner. The polishing endpoint for all specimens was defined as the achievement of a high-shine surface appearance, characterized by a visually smooth, glossy, and scratch-free surface. Following polishing, specimen thicknesses were verified using a digital caliper, and specimens not meeting the predefined dimensional criteria were excluded from the study. The specimens were cleaned with deionized water in an ultrasonic cleaner (Steris Reliance Sonic 250, Mentor, OH, USA) for 15 min, after which the color and surface roughness measurements of the specimens were recorded prior to thermocycling.

Subsequently, all specimens underwent 5000 thermal cycles in a coffee solution, alternating between temperatures of 5 °C and 55 °C with a dwell time of 30 s in each bath, using a thermocycling machine (SD Mechatronic GmbH, Feldkirchen-Westerham, Germany). Thermocycling was deliberately performed in coffee rather than in distilled water followed by separate coffee immersion in order to simulate a combined aging challenge, reflecting repeated exposure of restorative materials to thermal fluctuations while in direct contact with a chromogenic beverage, such as habitual hot and cold coffee consumption. By using coffee as the cycling medium, thermal stress and continuous chromogen interaction were applied simultaneously, allowing assessment of material behavior under conditions that more closely approximate daily oral exposure. The transfer time between temperature baths was approximately 5 s, resulting in an effective cycle duration of approximately 70 s (30 s cold dwell + 5 s transfer + 30 s hot dwell + 5 s return transfer). Accordingly, the total thermocycling duration was approximately 350,000 s for 5000 cycles. During the thermocycling procedure, specimens were maintained within the thermocycler chambers and were not subjected to additional storage in a separate medium, ensuring continuous exposure to the coffee solution throughout the aging process. The coffee solution was prepared using a commercially available medium-roast filter coffee (Lavazza Qualità Rossa, Luigi Lavazza S.p.A., Turin, Italy), following a standardized protocol. Specifically, one tablespoon of ground coffee was brewed with 177 mL of boiling water using a filter coffee machine. To maintain consistent staining potential and chromogen concentration throughout the procedure, the coffee solution was renewed every 12 h [3, 5, 10].

After thermocycling, measurements were repeated. Each main group was subsequently divided into two subgroups (*n* = 20) according to the toothpaste used. Specimens were brushed with two different commercially available dentifrices: a whitening toothpaste (WT) containing hydrated silica and hydrogen peroxide (Colgate Optic White, Colgate-Palmolive, NY, USA), and a non-whitening toothpaste (NWT) containing hydrated silica only (Sensodyne Repair and Protect, Haleon, UK).

Toothbrushing simulation was performed using a computer-controlled toothbrush simulator (Lua BS12 Toothbrush Simulator, Lua Instruments, Istanbul, Turkey) under standardized conditions. CrossAction-type brush heads (Oral-B Pro 1000, Procter & Gamble, Leicester, UK) were used for all specimens. According to manufacturer specifications, these brush heads are equipped with 16° angled nylon filaments. To minimize variability related to bristle wear, toothbrush heads were replaced after every 2000 brushing cycles. Brushing was performed under a constant vertical load of 200 g (≈ 2 N) at a rate of 150 strokes per minute, using a back-and-forth linear motion with a stroke distance of 10 mm, within the operating speed and displacement limits of the brushing simulator, for a total of 10,000 brushing cycles, corresponding to approximately one year of clinical toothbrushing. The applied vertical load was delivered axially through the toothbrush handle, directly above the brush head, ensuring uniform force distribution to the bristles in contact with the specimen surface [18].

Brushing was performed using a toothpaste slurry prepared by mixing 250 g of toothpaste with 1 L of distilled water, which was continuously supplied during brushing to simulate intraoral conditions and ensure uniform exposure of the specimen surfaces [18, 29].

Based on publicly available Relative Dentin Abrasivity (RDA) reference charts and manufacturer information, both toothpastes fall within the moderate abrasivity range (approximately 70–100). The whitening toothpaste has been reported to exhibit RDA values closer to the upper end of this range, whereas the non-whitening formulation is designed to provide effective cleaning while maintaining lower-to-moderate abrasivity, consistent with its indication for dentin hypersensitivity. Both dentifrices were intentionally selected to represent commercially available formulations with comparable, clinically acceptable abrasivity.

### Color measurement

All color measurements were performed under standardized conditions by a single, blinded operator—unaware of the treatments applied to the specimens—using a clinical spectrophotometer (VITA Easyshade Advance, Zahnfabrik, Bad Säckingen, Germany). Measurements were taken relative to the D65 standard illuminant against a standard white background, according to the Commission Internationale de l’Éclairage (CIE) L*a*b* color system and following the CIEDE2000 (ΔE₀₀) color difference formula.

Before each measurement session, the spectrophotometer was calibrated according to the manufacturer’s instructions using the device’s ceramic calibration block. For each specimen, three consecutive measurements were obtained at the same central surface area, and the mean L*, a*, and b* values were recorded to minimize measurement variability.

The L* coordinate represents luminance on a scale from 0 (black) to 100 (white), while the a* coordinate indicates the chromatic axis ranging from green (−) to red (+), and the b* coordinate represents the chromatic axis ranging from blue (−) to yellow (+).

Color coordinates of all specimens were recorded at three time points: baseline (T0), after coffee thermocycling (T1), and following the toothbrushing simulation (T2). Prior to each color measurement, specimens were ultrasonically cleaned for 15 min (Steris Reliance Sonic 250, USA), rinsed, and subsequently air-dried for 30 s. Color measurements were performed after this standardized drying period, not immediately after rinsing, in order to ensure consistency across all measurement time points and to eliminate the influence of surface moisture. Color differences (ΔE₀₀) were subsequently calculated for the intervals T1–T0 and T2–T1 using the CIEDE2000 formula:

$$\Delta\mathrm{E2000}=\sqrt{\left(\frac{\Delta\mathrm{L}^\prime}{K_{L}S_{L}}\right)^{2}+\left(\frac{\Delta\mathrm{C}^\prime}{K_{C}S_{C}}\right)^{2}+\left(\frac{\Delta\mathrm{H}^\prime}{K_{H}S_{H}}\right)^{2}+\, R_{T}\left(\frac{\Delta\mathrm{C}^\prime}{K_{C}S_{C}}\right)\left(\frac{\Delta\mathrm{H}^\prime}{K_{H}S_{H}}\right)}$$

### Surface roughness

All surface roughness measurements were performed under standardized conditions by a single, blinded operator, who was unaware of the treatment protocols applied to the specimens. Surface roughness was quantified using the arithmetic mean roughness parameter (Ra) with a contact profilometer (Perthometer M2, Mahr GmbH, Göttingen, Germany) and a tracing length of 4 mm. To preserve uniformity, the specimens were placed on a specific jig to assure their position and stability during the testing process. Five measurements were taken from randomly selected points on the surface of each specimen, and the average of these readings was considered as the representative Ra value for the respective sample. The Ra parameter was recorded at three time points: baseline (T0), after thermocycling in the coffee solution (T1), and after the toothbrushing simulation (T2), in order to evaluate the changes associated with thermal aging and mechanical abrasion. Ra was selected as the primary roughness parameter due to its widespread use in the literature and its clinical relevance in evaluating surface roughness of restorative materials, particularly with respect to clinically relevant thresholds related to plaque retention.

### Scanning Electron Microscopy (SEM) analysis

One randomly selected specimen from each group was subjected to scanning electron microscopy (SEM) analysis at the baseline and after the toothbrushing procedure. Prior to examination, the specimens were ultrasonically cleaned, air-dried, and sputter-coated with gold. The specimens were then mounted on aluminum stubs and placed in a charge-reduction specimen holder.

SEM evaluation was performed using a scanning electron microscope (JSM-6400 SEM, JEOL, Tokyo, Japan) operating in backscattered electron mode under low-vacuum conditions. Micrographs were acquired at an accelerating voltage of 5 kV and at a magnification of ×1000 to qualitatively assess surface topography and morphological changes induced by toothbrushing.

### Statistical analysis

The analyses were performed using IBM SPSS Statistics version 23. For numerical variables, the median, minimum, maximum, mean, and standard deviation values were reported. The normality of distribution for numerical variables was assessed using the Shapiro-Wilk test and box-plot graphs. The Mann-Whitney U test was used to evaluate differences in numerical variables between two independent groups. For comparisons involving more than two independent groups, the Kruskal-Wallis test was employed. When the Kruskal-Wallis test indicated a statistically significant overall difference, pairwise comparisons were conducted using the Dunn-Bonferroni post hoc test. A p-value of less than 0.05 was considered statistically significant.

## Results

### Color stability

The mean L*, a*, and b* values of all materials at baseline (T0), after coffee thermocycling (T1), and after brushing (T2) are presented in Table 2.

**Table 2:** Mean ± SD L*, a*, and b* values of the materials at baseline (T0), after coffee thermocycling (T1), and after brushing (T2)

GROUP	T0			T1			T2		
	L	a	b	L	a	b	L	a	b
IE	86.69 ± 0.84	2.51 ± 0.15	26.85 ± 0.53	86.57 ± 0.30	2.61 ± 0.10	27.02 ± 0.20	86.32 ± 0.88	2.50 ± 0.28	26.93 ± 0.68
TS	80.55 ± 0.74	2.77 ± 0.30	40.56 ± 1.49	80.34 ± 0.21	2.94 ± 0.11	40.45 ± 0.96	80.24 ± 0.52	2.93 ± 0.21	40.45 ± 1.02
VE	84.12 ± 1.33	2.81 ± 0.20	30.95 ± 1.17	84.65 ± 0.55	2.96 ± 0.10	30.64 ± 0.27	84.07 ± 0.72	3.01 ± 0.15	30.90 ± 0.65
SH	84.17 ± 1.22	1.71 ± 0.09	27.30 ± 0.62	85.45 ± 0.37	2.16 ± 0.07	27.37 ± 0.32	83.75 ± 0.49	2.05 ± 0.08	27.13 ± 0.29

*IE* IPS e.max, *TS* CEREC Tessera, *VE* Vita Enamic, *SH* Shofu

### Staining after coffee thermocycling (T1–T0)

**Fig. 1:**
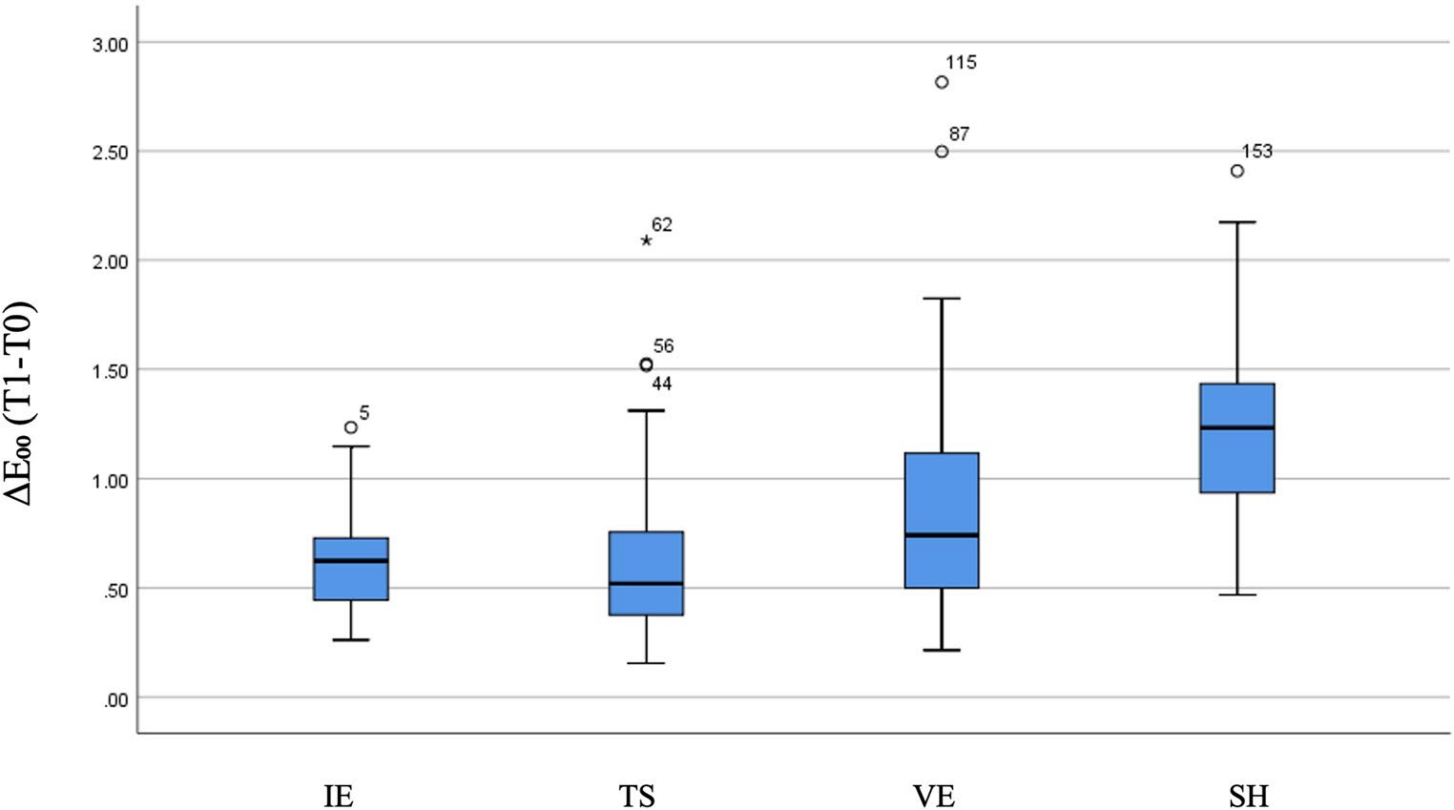
Box plots illustrating the distribution of ΔE₀₀ values reflecting color stability (T1–T0) across the groups, with medians shown. IE, IPS e.max; TS, CEREC Tessera; VE, Vita Enamic; SH, Shofu

There was a statistically significant overall difference among the groups in terms of color stability, as assessed by ΔE₀₀ values (thermocycling–baseline) (*p* < 0.001). According to the pairwise comparison test results, the median ΔE₀₀ value indicating reduced color stability in the Shofu group was significantly higher than that of the other three groups (Fig. 1). No significant differences were observed among the remaining three groups (Table 3).

**Table 3:** The comparison of groups in terms of color stability based on ΔE₀₀ values (T1–T0)

Variable	Group	Median (Min, Max)	Mean ± SD	Test Statistic	*p*-value	Significant Differences
ΔE₀₀ (T1-T0)	IE	0.624 (0.261, 1.235)	0.615 ± 0.226	46.53	< 0.001	TS-SH IE-SH VE-SH
	TS	0.520 (0.154, 2.090)	0.645 ± 0.411			
	VE	0.741 (0.215, 2.816)	0.892 ± 0.571			
	SH	1.234 (0.468, 2.409)	1.222 ± 0.421			

*IE* IPS e.max, *TS* CEREC Tessera, *VE* Vita Enamic, SH Shofu

Perceptibility threshold (PT, ΔE₀₀=0.8); Acceptability threshold (AT, ΔE₀₀=1.8)

### Staining after brushing (T1–T2)

**Fig. 2:**
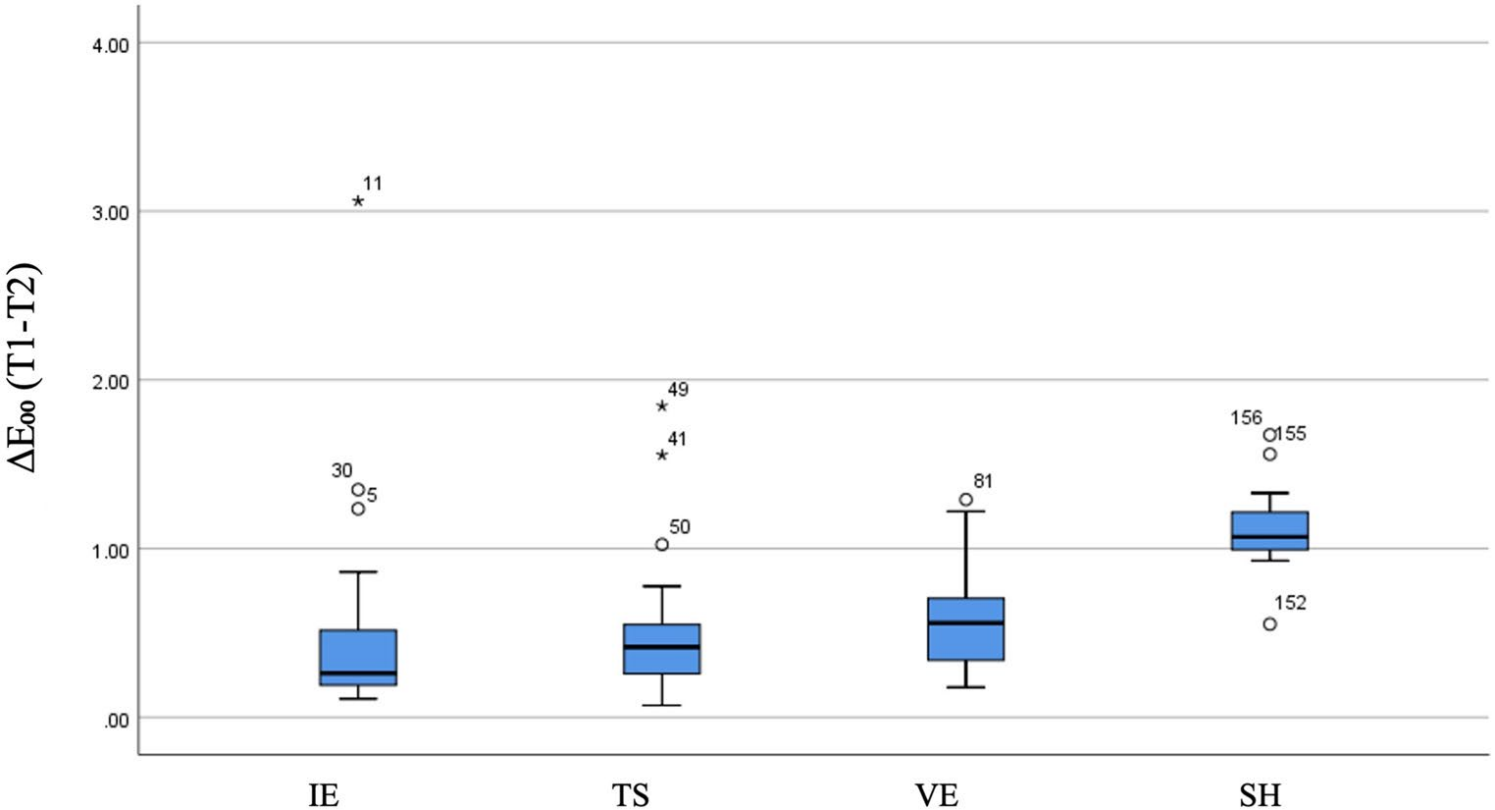
Box plots illustrating the distribution of ΔE₀₀ values reflecting color stability (T1–T2) across the groups, with medians shown. IE, IPS e.max; TS, CEREC Tessera; VE, Vita Enamic; SH, Shofu

A statistically significant difference was also identified among the material groups regarding color stability, as assessed by ΔE₀₀ values after brushing coffee-stained specimens (*p* < 0.001). The highest median ΔE₀₀ value, indicating reduced color stability, was again observed in the SH material (1.069), which was significantly higher than that of the other three material groups. Furthermore, a significant difference was detected between the median values of the IE (0.261) and VE (0.558) groups (Table 4; Fig. 2).

**Table 4:** The comparison of groups in terms of color stability based on ΔE₀₀ values (T1-T2)

Variable	Group	Median (Min, Max)	Mean ± SD	Test Statistic	*p*-value	Significant Differences
ΔE₀₀ (T1-T2)	IE	0.261 (0.109, 3.060)	0.444 ± 0.513	74.635	< 0.001	IE-VE IE-SH TS-SH VE-SH
	TS	0.416 (0.070, 1.845)	0.487 ± 0.346			
	VE	0.558 (0.177, 1.290)	0.567 ± 0.270			
	SH	1.069 (0.552, 1.674)	1.115 ± 0.191			

*IE* IPS e.max, *TS* CEREC Tessera, *VE* Vita Enamic, *SH* Shofu

Perceptibility threshold (PT, ΔE₀₀=0.8); Acceptability threshold (AT, ΔE₀₀=1.8)

When examining the effects of different toothpastes on color stability after brushing, no statistically significant difference was found between the two toothpastes for any of the material groups (*p* > 0.05) (Table 5).

**Table 5:** Comparison of toothpastes within groups regarding color stability based on ΔE₀₀ values (T1–T2)

Variable	Group	Toothpastes	Median (Min, Max)	Mean ± SD	Test Statistic (U)	*p*-value
ΔE₀₀ (T1-T2)	IE	WT	0.246 (0.109, 3.060)	0.490 ± 0.667	185.00	0.698
		NWT	0.261 (0.114, 1.349)	0.397 ± 0.301		
	TS	WT	0.489 (0.07, 1.845)	0.582 ± 0.435	136.00	0.086
		NWT	0.346 (0.186, 0.776)	0.391 ± 0.192		
	VE	WT	0.558 (0.177, 1.29)	0.568 ± 0.272	199.00	0.989
		NWT	0.545 (0.253, 1.22)	0.567 ± 0.275		
	SH	WT	1.071 (0.928, 1.329)	1.123 ± 0.137	163.00	0.327
		NWT	1.009 (0.552, 1.674)	1.107 ± 0.237		

*IE*, IPS e.max, *TS* CEREC Tessera, *VE* Vita Enamic, *SH* Shofu, *WT* Whitening Toothpaste, *NWT* Non-Whitening Toothpaste

Perceptibility threshold (PT, ΔE₀₀=0.8); Acceptability threshold (AT, ΔE₀₀=1.8)

### Surface roughness

**Fig. 3:**
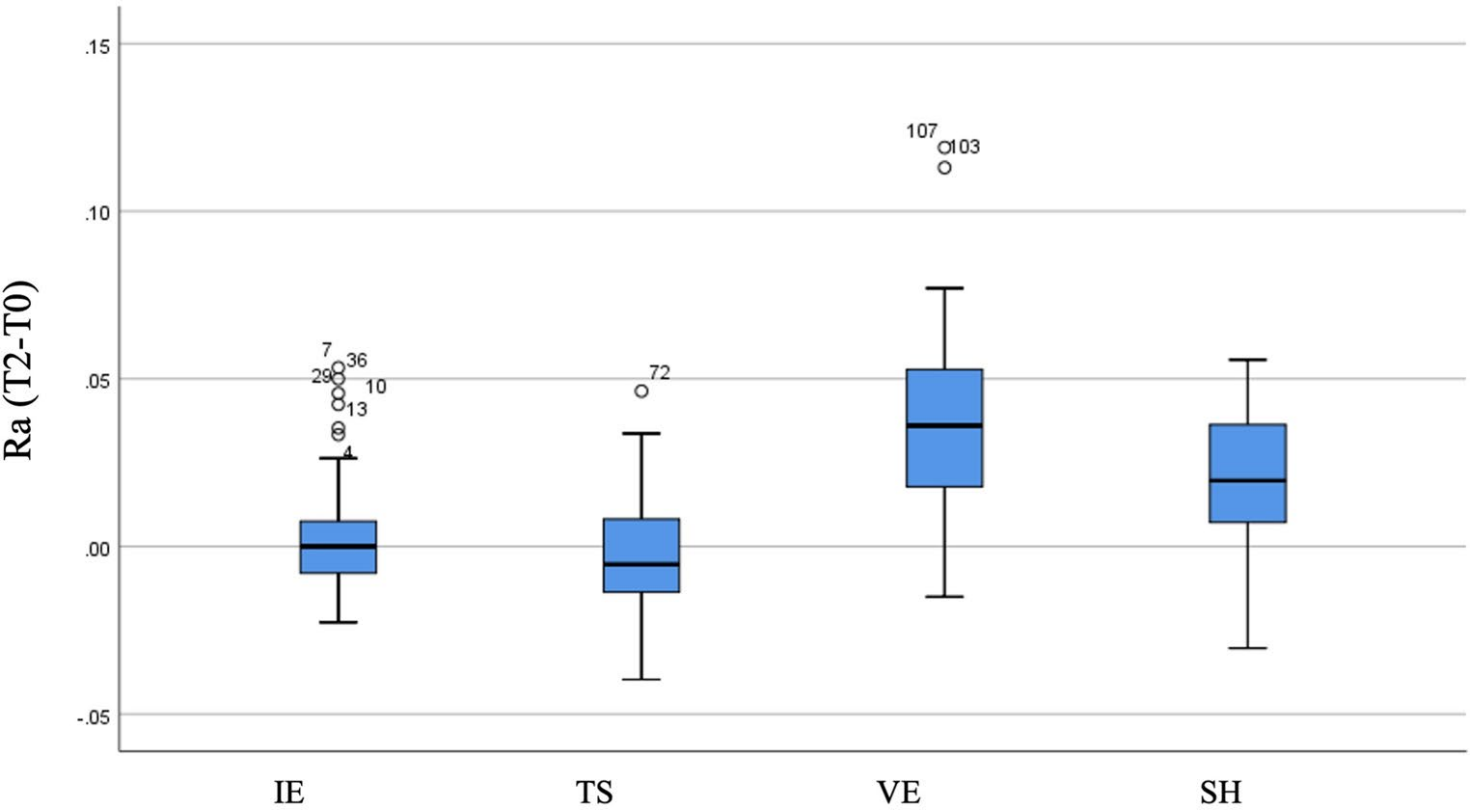
Box plots illustrating the distribution of changes in the Ra surface roughness parameter (T2–T0) across the groups, with medians shown. IE, IPS e.max; TS, CEREC Tessera; VE, Vita Enamic; SH, Shofu

There was a statistically significant difference among the material groups in terms of changes in the Ra parameter (T2-T0) following the brushing simulation (*p* < 0.001). Pairwise comparisons revealed that the increase in Ra values for VE (0.036) and SH (0.020) was significantly greater than that for IE (0.000) and TS (-0.005). No significant differences in Ra changes were found between the IE and TS groups or between the VE and SH groups (Table 6; Fig. 3). The absolute mean Ra values measured after brushing (T2) were 0.055 μm for IE, 0.045 μm for TS, 0.098 μm for VE, and 0.066 μm for SH.

**Table 6:** Comparison of groups in terms of changes in the Ra surface roughness parameter (T2–T0)

Variable	Group	Median (Min, Max)	Mean ± SD	Test Statistic	*p*-value	Significant Differences
Ra (T2-T0)	IE	0.000 (-0.023, 0.053)	0.004 ± 0.019	55.161	< 0.001	TS-SH TS-VE IE-SH IE-VE
	TS	-0.005 (-0.040, 0.046)	-0.003 ± 0.020			
	VE	0.036 (-0.015, 0.119)	0.038 ± 0.028			
	SH	0.020 (-0.030, 0.056)	0.019 ± 0.020			

*IE* IPS e.max, *TS* CEREC Tessera, *VE* Vita Enamic, *SH* Shofu

When evaluating the effect of different toothpastes on surface roughness, no statistically significant difference in surface roughness values was found between the WT and NWT for any of the four material groups tested (*p* > 0.05) (Table 7).

**Table 7:** Comparison of toothpastes within groups in terms of changes in the Ra surface roughness parameter (T2–T0)

Variable	Group	Toothpastes	Median (Min, Max)	Mean ± SD	Test Statistic (U)	*p*-value
Ra (T2-T0)	IE	WT	0.000 (-0.023, 0.053)	0.005 ± 0.021	196.50	0.925
		NWT	0.000 (-0.018, 0.050)	0.004 ± 0.019		
	TS	WT	-0.006 (-0.040, 0.034)	-0.006 ± 0.020	170.00	0.429
		NWT	-0.001 (-0.032, 0.046)	0.000 ± 0.019		
	VE	WT	0.039 (0.009, 0.077)	0.038 ± 0.020	180.50	0.602
		NWT	0.031 (-0.015, 0.119)	0.037 ± 0.035		
	SH	WT	0.020 (-0.030, 0.049)	0.018 ± 0.020	193.50	0.862
		NWT	0.020 (-0.019, 0.056)	0.020 ± 0.021		

*IE* IPS e.max, *TS* CEREC Tessera, *VE* Vita Enamic, *SH* Shofu, *WT* Whitening Toothpaste, *NWT* Non-Whitening Toothpaste

### SEM analysis

SEM images demonstrated material-dependent surface changes after simulated toothbrushing. At baseline (T0), all materials exhibited smooth and homogeneous surfaces with polishing-related features.

**Fig. 4:**
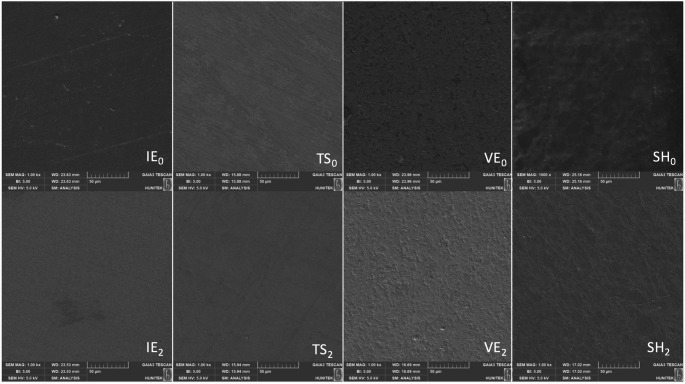
Representative SEM images (×1000 magnification) of CAD/CAM restorative materials at baseline (T0) and after simulated toothbrushing (T2). IE, IPS e.max; TS, CEREC Tessera; VE, Vita Enamic; SH, Shofu

Glass-ceramic materials (IE and TS) maintained surface integrity after brushing (T2), showing only minor smoothing of polishing marks and no evident surface damage. In contrast, resin-containing materials (VE and SH) exhibited more pronounced surface alterations after brushing, including increased roughness, micro-pits, and irregular wear patterns. These changes were more evident in VE and SH specimens, indicating greater susceptibility of the resin matrix to brushing-induced abrasion (Fig. 4).

## Discussion

The purpose of this laboratory study was to investigate the effects of toothbrushing with different dentifrice formulations on the color stability and surface characteristics of restorations stained with coffee. The null hypothesis was that coffee immersion and brushing with different toothpaste formulations would not cause any significant differences in the color stability or surface roughness of the tested materials. Based on the findings, this null hypothesis was partially rejected. Statistically significant differences were observed among the materials with respect to color stability after coffee thermocycling and changes in surface roughness after brushing. However, the part of the hypothesis stating that whitening and non-whitening toothpaste formulations would have no effect was accepted, as they did not produce statistically significant differences in color or surface roughness.

Color stability is a critical factor for the long-term esthetic success of restorative materials [26]. In most previous studies, IPS e.max CAD has been the preferred choice for evaluating the optical and mechanical performance of glass-ceramics, owing to its well-established clinical background. However, in the present study, CEREC Tessera, an ‘advanced lithium disilicate’ representing a relatively new generation of CAD/CAM glass-ceramics, was also included. The purpose of incorporating TS was to investigate whether a novel material with a modified microstructure and firing protocol would exhibit comparable or distinct responses to staining and brushing challenges [10, 16, 17].

The present findings demonstrated that resin-based materials (SH and VE) exhibited lower color stability after coffee thermocycling compared with glass-ceramic materials (IE and TS). Specifically, the resin nanoceramic SH exhibited the greatest reduction in color stability after coffee thermocycling, as indicated by the highest ΔE₀₀ value (ΔE₀₀=1.234). This value is above the perceptibility threshold (PT, ΔE₀₀=0.8) defined by Paravina et al., but below the acceptability threshold (AT, ΔE₀₀=1.8) [30]. After brushing, only Shofu continued to exhibit a ΔE value above the perceptibility threshold. This observation suggests that SH may be more susceptible to extrinsic factors compared with the other tested materials. Depending on the material used clinically, this condition may require repolishing or, in some cases, replacement of the restoration. This finding is consistent with previous studies indicating that the polymer matrices of resin-containing materials exhibit lower color stability due to their greater interaction with water and oral fluids [3, 5]. The reduced color stability observed after coffee exposure can be attributed not only to its strong pigmentation but also to its temperature and acidity, which facilitate both surface and subsurface penetration of chromogenic agents. Polar colorants present in coffee may infiltrate resin matrices and be retained within the material structure over time, thereby adversely affecting color stability [14]. Accordingly, the thermocycling protocol was designed to simulate temperature fluctuations around the physiological oral temperature, thereby promoting clinically relevant interactions between thermal conditions and staining. From a clinical perspective, the combination of coffee immersion and thermocycling represents a relevant experimental approach, as it reflects dietary staining and temperature variations commonly encountered in the oral environment. Coffee is one of the most frequently consumed beverages worldwide and is well known for its strong staining potential due to its high content of chromogenic agents. Repeated thermal changes associated with the intake of hot and cold beverages may further facilitate the penetration of colorants into restorative materials. Within this context, the reduced color stability observed in resin-containing materials aligns with previous studies demonstrating their higher water absorption potential and increased susceptibility to chromogen uptake [3, 5]. Stamenkovic et al. reported that resin nanoceramics (RNCs) and polymer-infiltrated ceramic networks (PICNs) exhibit lower color stability compared to lithium silicate-based ceramics when exposed to staining solutions like coffee and red wine [3]. The water absorption potential and hydrophilicity of the resin matrix facilitate the penetration of coffee’s yellow pigments into the material’s microstructure, thereby negatively influencing color stability [9]. This is further supported by findings showing that resin-based Cerasmart exhibited higher ΔE₀₀ values, indicating lower color stability, compared with zirconia-reinforced lithium silicate ceramics [21].

A remarkable finding of this study was that whitening and non-whitening toothpastes did not produce a statistically significant difference in terms of color stability or surface roughness across all tested materials. This suggests that the abrasiveness of whitening toothpastes may not always lead to greater surface degradation. Mascaro et al. noted that the conventional and whitening toothpastes they used both fell within the medium abrasiveness range, which could explain the lack of substantial differences in their findings [5]. Similarly, Al-Angari et al. reported that the effectiveness of whitening toothpastes in removing stains is primarily attributed to the mechanical action of brushing and the polishing effect of their abrasive particles [31]. However, a study found that whitening toothpastes containing silica and charcoal can significantly increase the surface roughness of resin-based nanoceramics [21]. Therefore, while the absence of a significant difference in this study may be explained by similar abrasive properties of the dentifrices or a minimal effect of the whitening agent, it should be noted that the type of abrasive particle can yield different results depending on the material.

The surface smoothness of a restoration is important for its longevity, as it reduces plaque accumulation and extrinsic staining [24, 32]. This study found that polymer-containing materials showed a significantly greater increase in surface roughness after brushing compared to glass-ceramics. This result indicates that the polymer matrix is more susceptible to mechanical abrasion than a pure ceramic structure. Flury et al. demonstrated that resin-based materials with lower hardness and modulus of elasticity are more prone to degradation from factors such as brushing and water storage [22]. In the present study, although polymer-containing materials exhibited a greater increase in surface roughness after brushing compared with glass ceramics, the final Ra values remained limited and below the commonly cited plaque retention threshold of approximately 0.2 μm. Therefore, despite the presence of statistically significant differences, the observed final Ra values are unlikely to be clinically relevant in terms of increased plaque accumulation. Similarly, de Andrade et al. reported that resin-containing materials exhibited the highest Ra values after brushing and, in line with the findings of the present study, demonstrated that the Ra value of the VE remained below the 0.2 μm threshold associated with bacterial plaque retention [24].

Surface finishing procedures also have a significant impact on the long-term performance of materials. While glazing is often preferred to provide a smooth surface, some studies have shown that mechanical polishing can yield superior results [11]. Brito et al. found that for all ceramics tested, mechanical polishing provided a significantly smoother surface (lower Ra values) and better color stability (lower ΔE values) compared to glazing [11]. Conversely, Garza et al. stated that a protective glaze layer on lithium disilicate increased its resistance to changes in color stability [23]. Another study found that glazed Celtra Duo exhibited reduced color stability compared with its unglazed counterpart, attributing this effect to abrasion of the glaze layer during aging [21]. These findings suggest that the protective effect or stability of a glaze layer can vary depending on the base material, the type of glaze, the firing protocol, and the oral environmental conditions it is exposed to.

The results of this laboratory study should be interpreted with consideration of its limitations. Laboratory conditions cannot fully replicate the complex dynamics of the oral environment, such as pH fluctuations, temperature changes, salivary enzymes, and occlusal forces [5, 25]. The 10,000 brushing cycles used represent a simulation of clinical use (e.g., 1 year), but there is no universal standard for this protocol across studies [18, 25]. As the TS material used in this study is new to the literature, the number of existing publications available for comparison of findings is limited [10, 17]. Furthermore, flat-surfaced specimens were used, which do not mimic the complex anatomical forms of restorations [25]. Only coffee was used as a staining agent; other common beverages such as red wine or tea could produce different results [9]. Future research should focus on evaluating the long-term effects of different dietary staining agents and toothbrushing protocols on a broader range of CAD/CAM restorative materials under simulated and clinical conditions.

## Conclusion

Within the limitations of this laboratory study, it was concluded that the long-term esthetic stability of restorations is strongly influenced by the intrinsic properties of the material rather than the abrasivity of the toothpaste used. Although statistically significant differences in color stability were observed among the tested materials, the ΔE₀₀ values were generally below the commonly accepted human perceptibility threshold, indicating limited clinical relevance. Glass-ceramics demonstrated superior color stability and greater resistance to brushing-related surface deterioration, underscoring their reliability for patients with high exposure to chromogenic beverages. Resin-based restorative materials, while offering favorable mechanical properties, may require more frequent maintenance or repolishing to preserve their appearance. Given the compositional and structural diversity of resin-based CAD/CAM materials, the present findings should be limited to the specific materials investigated in this study. These insights emphasize the importance of evidence-based material selection and encourage further clinical investigations to validate laboratory findings under real oral conditions. 

## Data Availability

The data supporting the findings of this study are available upon reasonable request from the corresponding author.
